# The Annual Meeting

**Published:** 1918-06-22

**Authors:** 


### ROYAL BRITISH NURSES' ASSOCIATION.

### THE ANNUAL MEETING.

The annual meeting of this Association was held at the rooms of the Medical Society of London on Thursday, June 13, under the presidency of H.R.H. Princess Christian. There was a large attendance.

The Annual Report for last year was presented by the Medical Hon. Secretary. This Report recalled the special meeting last December to receive the Report of the negotiations with the College of Nursing, Ltd., and stated that during the year fifty-four applications for registration had been accepted and sixty-three nurses had been elected members. The number was smaller than in previous years, because while negotiations concerning amalgamation with the College of Nursing were in progress it was understood that it would make little difference on which register a nurse placed her name. Also it had transpired that the propaganda for the College in one of the weekly nursing papers led many nurses to believe that the register of their own Association had been closed. Another cause for the smaller number of applications was that neither of the half-yearly lists from the Australian branch had reached the office; presumably they had been lost. Referring to the non-acceptance of the Privy Council alterations in the Supplemental Charter, the Report said the Council are of opinion that in any Bill for State Registration of nurses three cardinal principles should be embodied: (1) a uniform curriculum; (2) a minimum standard of three years' training; (3) a one-portal examination. The alterations made in the Supplemental Charter did not safeguard those principles. Regret was expressed at the resignation of Mr. Comyns Berkeley as hon. treasurer, and the Report paid a tribute to him for his valuable services. In the obituary special reference is made to the labours of the late Dr. Bezly Thorne, and fitting regret is also extended in regard to the decease of Mrs. Milton (Lady Consul at Cairo) and Miss Dawson, who died on the *Salta* when that vessel was lost. It was hoped that Parliament would soon recognise the splendid work of nurses by passing before long a Bill for the State Registration of fully-trained nurses; this would not only secure to them economic independence, but would protect the public, raise the standard of nursing, and improve the fully-trained nurse's status.

Mr. Herbert J. Paterson, on presenting the Report, spoke of the most valuable work done for the Corporation by its Secretary, Miss Macdonald; she had always responded most cheerfully to the extra demands made on her time and energies. Since the Report was drawn up certain societies had become affiliated with the Corporation, as the direct result of a conference convened by the Royal President. These were the Matrons' Council of Great Britain and Ireland, the Society for the State Registration of Trained Nurses, the Scottish Nurses' Association, the Irish Nurses' Association, and the Fever Nurses' Association. By an agreement drawn up these bodies would have the right to nominate representatives to the Corporation's Council. It was also hoped that the Trained Nurses' Annuity Fund would amalgamate with that of the Corporation, and thus harmonise in an economical administration of funds. He concluded by urging nurses to be alive to their interests, and in securing their economic independence. He proposed the adoption of the Report.

Miss Kent seconded in a graceful speech of recognition of the work of Miss Macdonald and Mr. Paterson for the good of the profession. She reminded the meeting that British nurses were but part of that great confraternity, the International Council of Nurses, in twenty-three countries, which she always regarded as a great spiritual alliance, a view which received pathetic confirmation by the recent heroic self-sacrifice in times of great danger. The resolution was unanimously carried.

Dr. Kenneth Stewart, Hon. Treasurer, presented the financial statement, which shows £90 on the wrong side on the year's working, due largely to the extra expenditure caused by the negotiations and the detailed reports supplied to members to keep them in touch with everything which was going forward. He proposed its adoption. This was seconded by Miss Sinzininex and carried.

H.R.H. the President announced, as already published, that Princess Beatrice and Princess Arthur of Connaught had become vice-presidents of the Corporation, and also Sir Owen Philipps, through whose gifts and activities the Corporation had received £300. Nurses had got to fight, and Her Royal Highness had no fear that, having recognised that fact, they would come out on top.

It was announced that the thirty names on the ballot paper for positions on the Council had been accepted. In addition, vacancies on the Council were filled by the election of Miss Pearse, Miss Drakard, Miss Cave, and Dr. Eric Pritchard. 454 voting papers were sent in unaltered, twelve were altered—five were unsigned. The auditors were re-elected, H.R.H. adding a word of commendation to Mr. Pittman. A cordial vote of thanks was passed to the honorary officers, and by acclamation to H.R.H. Princess Christian for presiding.

